# Heparan Sulfate Inhibits Hematopoietic Stem and Progenitor Cell Migration and Engraftment in Mucopolysaccharidosis I*

**DOI:** 10.1074/jbc.M114.599944

**Published:** 2014-10-30

**Authors:** H. Angharad Watson, Rebecca J. Holley, Kia J. Langford-Smith, Fiona L. Wilkinson, Toin H. van Kuppevelt, Robert F. Wynn, J. Edmond Wraith, Catherine L. R. Merry, Brian W. Bigger

**Affiliations:** From the ‡ Stem Cell & Neurotherapies Group, Faculty of Medical and Human Sciences, University of Manchester, Manchester M13 9PT, United Kingdom,; § Stem Cell Glycobiology Group, Faculty of Physical and Engineering Sciences, University of Manchester, Manchester M13 9PL, United Kingdom,; ¶ Matrix Biochemistry Group, Department of Biochemistry, Radboud University Nijmegen Medical Center, 6500 HB Nijmegen, The Netherlands,; ‖ Bone Marrow Transplantation Unit, Royal Manchester Children's Hospital, Manchester M13 9WL, United Kingdom, and; ** Biochemical Genetics Unit, St. Mary's Hospital, Manchester M13 9WL, United Kingdom

**Keywords:** Animal Model, Bone Marrow, Hematopoietic Stem Cells, Heparan Sulfate, Lysosomal Storage Disease, Migration, Bone Marrow Transplant, CXCL12, Mucopolysaccharidosis I, Hurler

## Abstract

Mucopolysaccharidosis I Hurler (MPSI-H) is a pediatric lysosomal storage disease caused by genetic deficiencies in IDUA, coding for α-l-iduronidase. *Idua*^−/−^ mice share similar clinical pathology with patients, including the accumulation of the undegraded glycosaminoglycans (GAGs) heparan sulfate (HS), and dermatan sulfate (DS), progressive neurodegeneration, and dysostosis multiplex. Hematopoietic stem cell transplantation (HSCT) is the most effective treatment for Hurler patients, but reduced intensity conditioning is a risk factor in transplantation, suggesting an underlying defect in hematopoietic cell engraftment. HS is a co-receptor in the CXCL12/CXCR4 axis of hematopoietic stem and progenitor cell (HSPC) migration to the bone marrow (BM), but the effect of HS alterations on HSPC migration, or the functional role of HS in MPSI-H are unknown. We demonstrate defective WT HSPC engraftment and migration in *Idua*^−/−^ recipient BM, particularly under reduced intensity conditioning. Both intra- but especially extracellular *Idua*^−/−^ BM HS was significantly increased and abnormally sulfated. Soluble heparinase-sensitive GAGs from *Idua*^−/−^ BM and specifically 2*-O*-sulfated HS, elevated in *Idua*^−/−^ BM, both inhibited CXCL12-mediated WT HSPC transwell migration, while DS had no effect. Thus we have shown that excess overly sulfated extracellular HS binds, and sequesters CXCL12, limiting hematopoietic migration and providing a potential mechanism for the limited scope of HSCT in Hurler disease.

## Teaser

**Background:** Hematopoietic stem cell transplant in mucopolysaccharidosis I (MPSI) patients often results in graft failure.

**Results:** In mice with MPSI we link reduced hematopoietic engraftment post-transplant to accumulated overly-sulfated extracellular heparan sulfate.

**Conclusion:** Excess extracellular heparan sulfate alters cytokine gradient formation, restricting stem cell migration.

**Significance:** This provides a mechanistic insight into the observed engraftment difficulties seen in patients.

## Introduction

Mucopolysaccharidosis type I (MPSI)^2^ is an inherited metabolic disease caused by a deficiency of α-l-iduronidase (IDUA), which leads to accumulation of heparan sulfate (HS) and dermatan sulfate (DS). Hurler disease (MPSI-H) is the most severe form, presenting in infancy with symptoms of skeletal dysplasia, abnormal facies, organomegaly, cardiac and respiratory defects, severe neurological degeneration, and death (1). Attenuated forms of the disease have much less significant neurological involvement and improved lifespan.

There are two current clinical treatment modalities for MPSI. Pharmacological enzyme replacement therapy (ERT), which relies on mannose-6-phosphate mediated uptake and cross-correction of affected cells, has limited effect on bone and neurological manifestations, as the enzyme fails to cross the blood brain barrier. Thus, ERT is only licensed for use in attenuated forms of MPSI (2, 3). Hematopoietic stem cell transplantation (HSCT) is an attractive alternative therapy, relying on the ability of donor derived monocytes to traffic and engraft in the brain as microglial cells (4), releasing enzyme here for cross correction. However, while successful transplants have resulted in significantly improved patient outcomes in MPSI-H, with extended life and improved neurological function, successful stable donor chimerism is difficult to achieve, with 44% of patients requiring a second or third transplant (5, 6). Risk factors identified in bone marrow (BM) and umbilical cord blood transplantation include reduced intensity conditioning regimens and age of treatment, with earlier treatment resulting in improved clinical outcomes. Recently, the introduction of fully myeloablative busulfan/cyclophosphamide conditioning (5–7), has significantly improved transplant success rates to over 90% in some centers, but this requirement suggests an underlying engraftment defect in MPSI patients in one or all of hematopoietic stem cell maintenance, homing, or engraftment.

HS, which is stored to pathological excess in MPSI-H (8–10), is involved in all aspects of these processes, including signaling involving CXCL12 (11, 12), as well as the presentation of chemokines to ligands in haptotactic gradients (13, 14). HS is synthesized covalently linked to a core protein to form an HS proteoglycan (HSPG), which are found at the cell-matrix interface. HS is a long chain non-branching sulfated glycosaminoglycan (GAG), consisting of repeating disaccharide subunits of *N*-acetyl glucosamine (GlcNAc) and glucuronic acid (GlcA). The chain is modified in the Golgi apparatus during synthesis by the epimerization of a subset of GlcA residues to iduronic acid (IdoA) and the transfer of sulfates to the *N*-, 2-*O-*, 3-*O*-, and 6-*O*-positions, selectively altering regions of the chain to patches of modification, known as sulfated (S-) domains, separated by regions with little modification. Ultimately it is these modifications which determine the ability of the chain to bind different protein factors (15). Heparin is structurally similar to HS but lacks a domain structure, being very heavily modified along the entire chain.

CXCL12 via its cognate receptor CXCR4, is the primary mediator of hematopoietic stem and progenitor cell (HSPC) homing to the BM following transplantation (16). It is constitutively expressed by the BM, and expression is up-regulated following DNA damage and hypoxia, which both result from myeloablative transplant conditioning (17, 18). CXCL12 is presented to incoming cells via HS on the apical surface of endothelial cells, and cannot function *in vivo* without its HS binding site (19), which is structurally unique among cytokines, and exhibits a binding preference for regions containing 2-*O-*sulfation (20).

Here, we demonstrate that engraftment difficulties seen in the clinic are recapitulated in a mouse model of MPSI-H, and that the decrease in engraftment can be linked to a decrease in homing of transplanted cells to the BM. The BM of these mice contains significant amounts of stored HS, but rather than the HS being sequestered within the lysosomes, it is instead overwhelmingly located in the extracellular matrix of the BM stroma, making it available for protein interactions. The accumulated HS is rich in the *2-O*-sulfation modification preferred by CXCL12, while levels of CXCL12 are increased in the BM niche of affected mice. However, we demonstrate that rather than improving migration of HSPCs to the BM, the excess, highly sulfated HS is inhibiting migration toward CXCL12, resulting in homing and engraftment defects.

## EXPERIMENTAL PROCEDURES

### 

#### 

##### Mice

Donor and recipient mice differentially expressed CD45 epitopes using crosses of B6.SJL-*Ptprc^a^Pepc^b^*/BoyJ (CD45.1) (Jackson Laboratories), C57BL6 (CD45.2) and heterozygote CD45.1xCD45.2 F1 mice, generated on WT or B6.129-*Idua^tm1Clk^*/J (*Idua*^−/−^) backgrounds (21). Mice were maintained according to UK Home Office regulations with food and water *ad libitum*.

##### BM Transplantation

6–9-week-old recipient mice were maintained on acidified water and irradiated feed for 7 days prior to transplant. Mice were either lethally irradiated in two 5 Gy doses 4 h apart (full intensity), or myeloablated using 125 (full intensity) or 25 mg/kg (reduced intensity) Busulfan (Busilvex, Pierre Fabre), via intraperitoneal injection as previously described (22–24). Fresh whole bone marrow or lineage-depleted cells were delivered via tail vein 1 h post-irradiation or 24 h post-Busulfan. Mice were sacrificed at 18 h (whole BM migration), 36 h (lineage depleted migration) 6 weeks (short term engraftment), or 20 weeks (long term engraftment) and BM, peripheral blood, spleen recovered and analyzed by flow cytometry to measure donor chimerism as previously described (22).

##### Leukocyte Preparation

BM or blood was collected, washed and erythrocytes lysed using ammonium chloride lysis. Extracted leukocytes were re-suspended in PBS/2% FCS for lineage negative separation (Stem Cell Technologies) or Sca1^+^ separation (Miltenyi) using double LS column selection, according to manufacturer's recommendations. Enriched cell populations were resuspended in X-Vivo10 for migration and whole BM stored in at > −80 °C in 10% DMSO/40% FCS. For quantification of total BM, cells from only one femur and one tibia per mouse were extracted and counted. Colony-forming assays were performed in triplicate as described previously with 2 × 10^4^ cells/ml for BM and 1 × 10^5^ cells/ml for peripheral blood and counted 12 days later (22–24).

##### CXCL12 Quantification

Quantification of CXCL12 was performed using a quantitative immunosorbant assay kit (R&D Systems) on the soluble fraction of BM extracted from a single femur and tibia in a total volume of 200 μl of PBS, and centrifuged at 300 × *g* to remove cells.

##### Bone Marrow Stromal Cell Culture

BM cells were isolated and lysed as described, but with additional mechanical abrasion of the interior of the bones using a 29 G needle, to extract MSCs residing at the endosteum (25). Cells were cultured in α-MEM (Lonza) with 12.5% horse serum, 12.5% FCS, 0.1 μm hydrocortisone, 2 mm l-glutamine, and 50 units of penicillin/50 μg of streptomycin. Passage 5–15 cells were characterized by flow cytometry with the following expression profile: CD45^−^, CD11b^−^, Sca1^+^, CD44^hi^, CD105^+^.

##### In Vitro Migration Assays

Solutions of HS, DS, or selectively desulfated heparins (Iduron), or 0.1% gelatin (Sigma) was used to coat the insert of a transwell plate (Costar 6.5 mm well, 5 μm pore inserts). Prior to migration, excess coating solution was aspirated, and inserts were washed once in PBS. For migration toward CXCL12, migration medium (X-Vivo 10) ± 200 ng/ml CXCL12 (R&D) was added to the lower well (*n* = 8). For all assays, cells were counted in triplicate and resuspended at 1 × 10^7^ cells/ml for whole, unsorted BM, or 1 × 10^5^ for HSPC enriched populations, in migration medium (X-VIVO 10, Lonza/5% FCS. Cells in 100 μl migration medium were added to the insert and migrated for 4 h at 37 °C. For migration toward the soluble fraction of BM, the pelvis, femurs and tibias of each mouse were flushed into 1 ml of PBS, pooled, and centrifuged at 300 × *g* to remove cells. 500 μl of undiluted soluble fraction was added to the lower well of the migration plate.

For preparation of purified glycosaminoglycans, soluble BM fractions were collected as above, and processed as described previously (10). GAGs were freeze dried and resuspended in the relevant starting volume of PBS. A portion of *Idua*^−/−^ GAGs were digested with heparinases as described previously (10). 2 × 10^5^ lineage depleted BM cells/well were migrated in the presence of 2% FCS/6.25% GAGs in X-VIVO 10 toward CXCL12 (200 ng/ml) for 8 h at 37 °C across gelatin-coated 5-μm pore transwell inserts. Migrated and un-migrated cells were counted by flow cytometry (BD FACSCanto II) using CytoCount Control Beads (Brookhaven Instruments Ltd).

##### Flow Cytometry

All flow cytometry was carried out on a BD FacsCANTO II flow cytometer, using FacsDiva (BD), FlowJo (TreeStar) or Weasel (WEHI Institute) software for analysis. ToPro3 (Invitrogen) or 7AAD (BD Biosciences) were used as viability stains. For the identification of hematopoietic stem and progenitor populations, the following antibodies were used: Rat αMouse C-Kit FITC; Lineage Mixture (CD3e, CD11b, CD45R, TER-119, Ly6G, Ly6C) APC; Rat αMouse Sca-1 PE; Rat αMouse CD45 APC-Cy7 (all BD Biosciences). For the identification of mature hematopoietic lineages, the following antibodies were used: Rat αMouse CD11b PE-Cy7; Rat αMouse CD19; Rat αMouse CD3 PerCPCy5.5 (all BD Biosciences). For measurement of donor chimerism: Mouse αMouse CD45.1 PE; Mouse αMouse CD45.2 (BD Biosciences). For the characterization of MSCs: Rat αMouse C-Kit FITC; Rat αMouse CD11b PE-Cy7; Rat αMouse Sca-1 PE; Rat αMouse CD45 APC-Cy7; Rat αMouse CD44 PerCP Cy5.5; Rat αMouse Sca-1 PE-Cy7 (BD Biosciences), and Rat αMouse CD105 FITC (R&D Systems). For the analysis of HS, the following phage display scFv antibodies were used: HS4C3, RB4EA12 and MPB49 as described previously (26, 27).

##### Immunofluorescence

WT and MPSI MSCs were cultured on Permanox chamber slides (Lab-Tek) and fixed with 4% paraformaldehyde, staining as described previously (27). Where required, slides were treated with 2 mIU heparinase I, II, and III in PBS for 1 h at room temperature prior to permeabilization. Antibodies used were LAMP2 (University of Iowa), 10E4 and, 3G10 (both Seikagaku), rat-Alexa546, mouse IgM-Alexa488, and mouse IgG-Alexa488 (all Invitrogen). Images were collected using a Nikon C1 confocal on an upright 90i microscope with a 60× oil immersion objective, with z-stacked images collected at 0.5-μm intervals. Images were processed in ImageJ (NIH/MacBiophotonics) and presented as z-projections at maximum intensity.

##### GAG/CXCL12 ELISA

BD Heparin Binding Plates (BD Biosciences) were used in all assays. HS, DS, and heparin (Iduron) were dissolved in PBS at concentrations of 0.05–12.8 μm based on approximate “average” disaccharide MWs. Plates were coated with GAG overnight at room temperature then washed with Wash Buffer (100 mm NaCl, 50 mm NaAc, 0.2% Tween20, pH 6.0), blocked with Wash Buffer +1% BSA (Sigma) for 90 min at 37 °C. Following incubation with 200 ng/ml CXCL12 in PBS for 90 min at 37 °C, wells were blocked with Wash Buffer/5% goat serum (Vector Labs) before adding 1:1000 polyclonal rabbit αhuman CXCL12 antibody (AbCam) for 1 h at room temperature. Wells were stained with biotinylated goat αrabbit IgG (Vector Labs,1:2000), amplified with Vectastain Elite ABC reagent (Vector Labs) and developed with OPD substrate (Sigma) as per manufacturer's instructions. Absorbance at 490 nm was measured using a Synergy HT Microplate Reader (BioTek).

##### 2-Aminoacridone (AMAC) Labeling for High Performance Liquid Chromatography Disaccharide Analysis

Leukocytes were extracted as described above. Supernatant from all wash and lysis steps was reserved for the extracellular niche fraction. The cell pellet was resuspended to form the cell fraction. Glycosaminoglycan chains were then purified, AMAC labeled and analyzed by RP-HPLC essentially as described previously (10). Because the efficiency of AMAC labeling varies according to specific disaccharides, the raw peaks were multiplied by the following correction factors determined by comparison to UV traces for individual disaccharides: UA2S-GlcNS6S, 1.25; UA-GlcNS(6S) 1.13; UA(2S)-GlcNS, 1.0; UA-GlcNS, 1.04; UA-GlcNAc(6S), 1.13; UA(2S)-GlcNAc, 0.97; UA-GlcNAc, 1.08. Quantification of the amount of HS contained within cell and matrix fractions was made by comparison of fluorescence with known quantities of HS.

##### Statistical Analysis

Comparisons of means was performed using either a two-tailed Student's *t* test, or one-way or 2-way ANOVA using JMP software (SAS Institute Inc.) appropriate to the number of means and variables compared. Post-hoc analysis used Tukey's multiple comparisons. *p* values of less than or equal to 0.05 were considered significant. Error bars refer to the standard error of mean.

## RESULTS

### 

#### 

##### Idua^−/−^ Mice Demonstrate a Defect in HSPC Engraftment and Bone Marrow Migration

**FIGURE 1.**
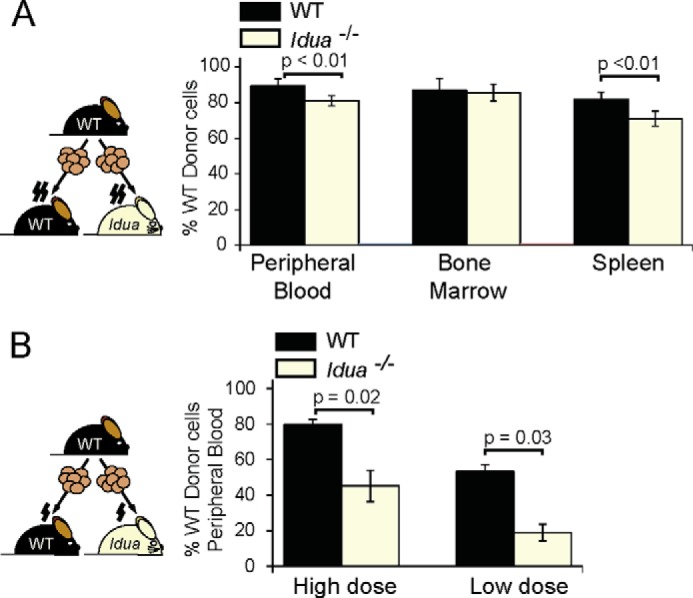
**There is a significant defect in engraftment in *Idua*^−/−^ mice under limiting conditions.** *A*, donor CD45+ cell chimerism in hematopoietic organs of *Idua*^−/−^ and WT mice 6 weeks after full intensity busulfan myeloablation and transplant of 2 × 10^6^ WT bone marrow cells (WT *n* = 4, *Idua*^−/−^ *n* = 6). *B*, donor CD45 cell chimerism in peripheral blood of *Idua*^−/−^ and WT mice 20 weeks after reduced intensity busulfan myeloablation and transplant of 1 × 10^7^ (high dose) or 2 × 10^6^ (low dose) WT bone marrow cells (*n* = 5). Two *Idua*^−/−^ recipients died prior to 20 weeks. Error bars represent standard error of mean (±S.E.).

To establish if an engraftment defect was present in MPSI and to mimic the treatment approach used in MPSI-H patients, we used HSCT in the *Idua*^−/−^ mouse model, which accumulates HS and DS and shares similar neuropathological features with MPSI patients (21, 28). Following full myeloablation of *Idua*^−/−^ or WT mice and transplantation with WT BM (23), full donor chimerism was achieved in all recipients at 6 weeks (Fig. 1*A*), although a small but statistically significant decrease in peripheral blood and spleen donor chimerism in *Idua*^−/−^ recipients was observed. We introduced limiting conditions for engraftment with reduced-intensity conditioning, resulting in a highly significant engraftment defect in *Idua*^−/−^ recipients at both high, or low cell doses (Fig. 1*B*).

**FIGURE 2.**
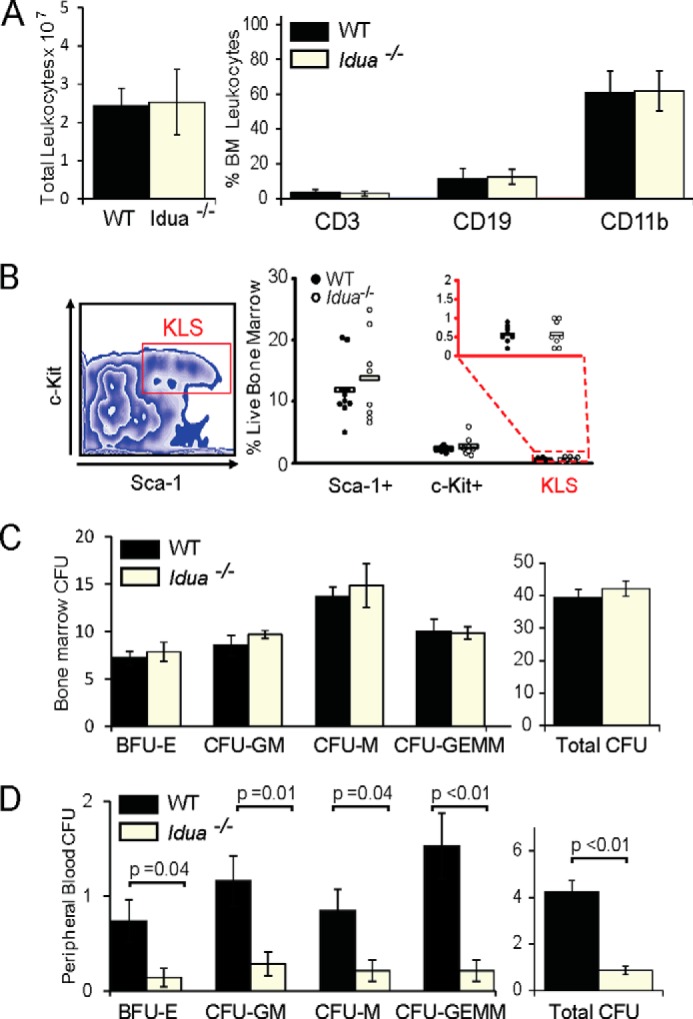
**BM Hematopoiesis is normal in *Idua*^−/−^ mice, but circulating HSPCs are significantly decreased.** *A*, total leukocytes isolated from a single femur and tibia of WT or *Idua*^−/−^ mice (*n* = 3–7). Relative proportion of mature hematopoietic lineages in bone marrow measured by flow cytometry (*n* = 9–10). Data from four independent experiments. *B*, flow cytometric gating and expression profile of C-Kit^+^, Lin^−^, Sca^−^1^+^(KLS) stem cell markers in WT and *Idua*^−/−^ mice. Proportion of KLS expressing BM cells (*n* = 8). Data from four independent experiments. *C*, colony-forming ability of BM or *D*, peripheral blood from age-matched WT and *Idua*^−/−^ mice and total colonies per plate (*n* = 7). Error bars represent ± S.E.

Previous analysis of mesenchymal stromal cells (MSCs) derived from bone marrow of MPSI-H patients, suggested that they were not able to maintain the colony-forming properties of HSCs as well as normal MSCs (29). This implied that the BM niche may be detrimental to hematopoiesis in MPSI. However, our analysis of total leukocytes in the BM suggested normal hemostasis in MPSI mice (Fig. 2*A*), supported by analysis of phenotypic markers of mature and naive hematopoietic populations (Fig. 2, *A* and *B*) and colony forming ability in the BM (Fig. 2*C*), both of which indicated no abnormality in the maintenance of mature or naive hematopoietic lineages in *Idua*^−/−^ BM. Interestingly, we did observe reduced colony forming units (CFU) in the peripheral blood of *Idua*^−/−^ mice (Fig. 2*D*), suggesting retention of HSPCs in the *Idua*^−/−^ BM compartment.

**FIGURE 3.**
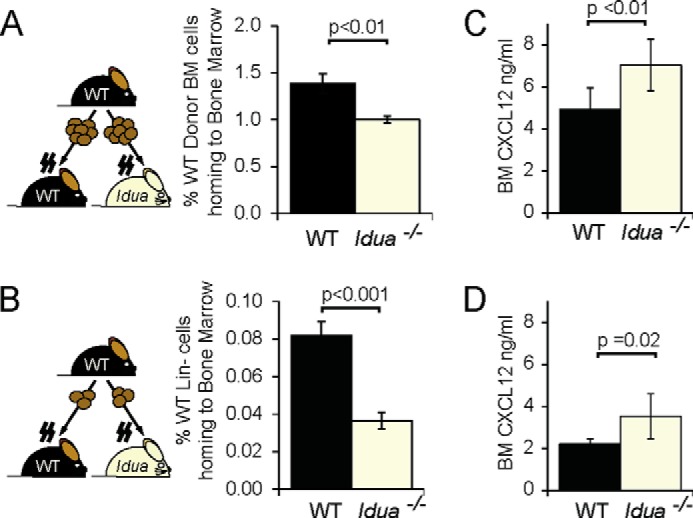
**Homing to the *Idua*^−/−^ BM is impaired despite increased niche CXCL12.** *A*, flow cytometric quantification of percentage of CD45-positive WT donor BM cells or *B*, lineage-depleted donor WT HSPCs migrating to the BM of WT or *Idua*^−/−^ recipients following lethal myeloablation and transplant with 2 × 10^7^ donor cells (*n* = 5–6). *C* and *D*, CXCL12 concentration in BM soluble fraction of WT and *Idua*^−/−^ mice, measured by quantitative ELISA (*n* = 5–15) in untreated mice (*C*) or 4 h after full myeloablation (*D*) (*n* = 5–6). Error bars represent ± S.E.

Short-term analysis of the ability of WT donor BM to migrate to the myeloablated bone marrow of recipients, revealed a significant decrease in the number of WT donor cells reaching the BM compartment in *Idua*^−/−^ recipients (Fig. 3*A*), that became more pronounced when using a donor BM lineage negative fraction enriched for HSPC (Fig. 3*B*). Reduced HSPC migration might suggest a decrease in the production of the chemokine CXCL12, known to be responsible for HSPC migration and retention in the *Idua*^−/−^ BM. However, analysis of CXCL12 concentration in the BM niche revealed significantly increased concentrations in *Idua*^−/−^ BM both before and after myeloablation (Fig. 3, *C* and *D*). Therefore the observed homing and engraftment defects are not due to decreased CXCL12. On the contrary, elevated levels of CXCL12 in the *Idua*^−/−^ BM (Fig. 3, *C* and *D*) may be responsible for the decreased levels of circulating HSPC observed in (Fig. 2*D*), supporting the hypothesis of increased retention of HSPCs in the BM niche.

##### Idua^−/−^ Bone Marrow Contains Excess Intra- and Extracellular HS in Non-lysosomal Locations That Is Rich in Sulfate Modifications, Particularly 2-O-Sulfation

CXCL12 relies on GAGs to form haptotactic gradients, which are thought to guide CXCL12 mediated migration (12). In addition, CXCL12 contains a unique HS binding site (11), permitting dimerization and CXCR4 receptor binding. CXCL12 binding is furthermore dependent on the level and pattern of sulfation of HS (11, 20, 30, 31). Thus we reasoned that alterations in the level and sulfation patterning of HS could alter the ability of HSPCs to migrate to CXCL12 gradients. Previous studies have been conflicting regarding quantities of HS stored and the level of sulfation in MPSI (32–34), and although we have identified similar patterns of increased and abnormally sulfated HS in tissue and serum in both *Idua*^−/−^ mice and MPSI patients (10), the BM has not been examined, nor the relative levels of intra- and extracellular HS quantified.

**FIGURE 4.**
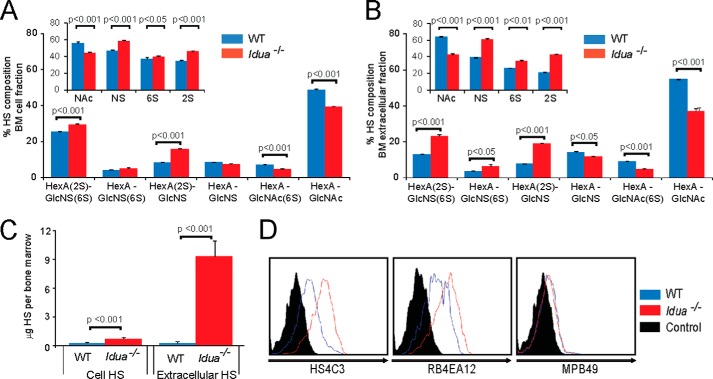
**Elevated amounts of abnormally sulfated HS in the intra- and extracellular bone marrow niche of *Idua*^−/−^ mice.** Disaccharide analysis of HS in the cellular (*A*) and extracellular (*B*) fraction of whole BM from *Idua*^−/−^ and WT mice was quantified by AMAC-tagged compositional analysis following complete digestion of the HS with combined heparinases. *Inset*: percentage contribution fractions for each sulfation position. *C*, total amount of HS in cellular and extracellular BM fractions. Amount of *Idua*^−/−^ BM is expressed as μg HS per bone marrow calculated from AMAC fluorescent readings compared with readings from measured amounts of HS standards. Data are compiled from a minimum of 5 separate mice of each genotype, separately digested, and analyzed at least twice. *D*, flow cytometric analysis of WT or *Idua*^−/−^ bone marrow-derived MSCs using HS-pattern specific antibodies. MPB49 is a non-HS binding control antibody. Data representative of eight independent experiments. Error bars represent ± S.E.

Biochemical analysis of the composition of HS in cellular and extracellular BM fractions, using reverse-phase HPLC and AMAC labeling (35), indicated that there is a significant increase in all sulfated disaccharides in both BM fractions in *Idua*^−/−^ mice, with the greatest increase being in overall levels of *2-O*-sulfate and *N-*sulfate modifications, and in particular, the HexA(2S)-GlcNS and HexA(2S)-GlcNS(6S) disaccharides (Fig. 4, *A* and 4*B*). Notably the proportion of disaccharides which are *N*-sulfated within *Idua*^−/−^ BM exceeds those which are *N*-acetylated. The most marked increase in HS accumulation was observed in the soluble extracellular BM fraction, not the cell fraction; a 33-fold increase in extracellular HS (average of 0.28 μg HS per WT BM compared with ∼9.4 μg of HS in *Idua*^−/−^) was visible compared with a 2.4-fold increase (average of 0.31 μg of HS in WT BM *versus* ∼0.73 μg in *Idua*^−/−^) in the cell fraction (Fig. 4*C*). Interestingly, WT BM has similar amounts of HS in the cell and extracellular fractions. However most of the excess stored HS in *Idua*^−/−^ BM is in the extracellular BM niche, (∼13-fold more than is stored in the cell fraction); the same location in which we found elevated levels of CXCL12 (Fig. 3, *C* and *D*). Analysis of CD45^−^, CD105^+^, CD44^+^ primary BM derived MSCs with phage-display scFv antibodies directed against specific three-dimensional sulfated HS epitopes, supports the disaccharide analysis, with increased binding of HS4C3 and RB4EA12 (Fig. 4*D*), both of which have been shown to recognize highly sulfated epitopes in HS, enriched for 6-*O*- and *N*-sulfation (36–38).

**FIGURE 5.**
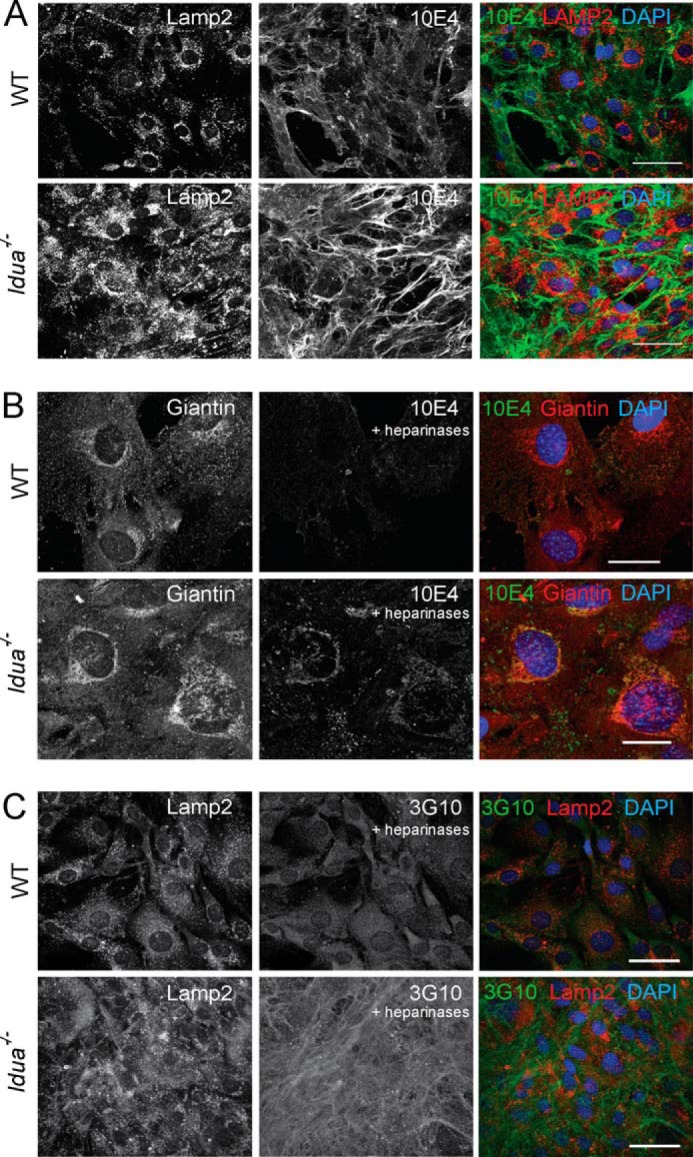
**HS is accumulated in non-lysosomal intracellular compartments including the Golgi.** *A*, confocal images of MSCs from WT or *Idua*^−/−^ mice stained for the lysosomal marker LAMP2 (*red*) and HS (10E4, *green*). Nuclei were stained with DAPI. Scale bar = 50 μm. *B*, WT and *Idua*^−/−^ MSCs were treated with heparinizes prior to permeabilization and subsequently stained with Giantin and 10E4. Scale bar = 25 μm. *C*, WT and *Idua*^−/−^ MSCs were treated with heparinizes prior to permeabilization and subsequently stained with Lamp2 and 3G10. Scale bar = 50 μm.

The 10E4 epitope lies in *N*-sulfated regions of the HS chain, but is destroyed through the combined and complete action of bacterial heparinases. Immunofluorescent staining of HS in MSCs confirmed a significant increase in cellular and matrix HS (Fig. 5*A*). Furthermore there was a marked absence of HS co-localized with LAMP2 in the lysosomal compartment, in agreement with our previous findings (10). Controlled heparinase treatment of *Idua*^−/−^ MSCs prior to permeabilization and staining, revealed non-lysosomal associated perinuclear 10E4 staining within the cell (Fig. 5*B*), co-localizing with the Golgi marker giantin. This staining was not strongly visible in WT cells (Fig. 5*B*). Therefore accumulated HS in BM MSCs is observed at the cell surface, in the ECM and in both lysosomal and non-lysosomal compartments. The 3G10 epitope is created by the action of heparinases, recognizing the unsaturated uronic acid stub remaining attached to proteoglycan cores (40). Thus it can provide information on the distribution of HS chains and therefore the proteoglycan cores within a tissue and potentially the number of HS chains (as only one 3G10 antibody can bind per chain). In WT cells, punctate 3G10 staining following heparinase digestion was seen across the surface of cells (Fig. 5*C*). However staining of *Idua*^−/−^ cells revealed extensive cell surface and ECM staining, with much of the epitopes remaining in tight bundles spanning across cell junctions (Fig. 5*C*). Again little co-localization with LAMP2 was apparent. The retention of the 3G10 epitope suggests that at least a proportion of the stored HS chains in the ECM of *Idua*^−/−^ cells are attached to proteoglycan cores rather than the HS chains being free, thus aiding retention of HS within the extracellular compartment.

##### Highly Sulfated HS from Idua^−/−^ Bone Marrow Inhibits CXCL12-mediated HSPC Migration

**FIGURE 6.**
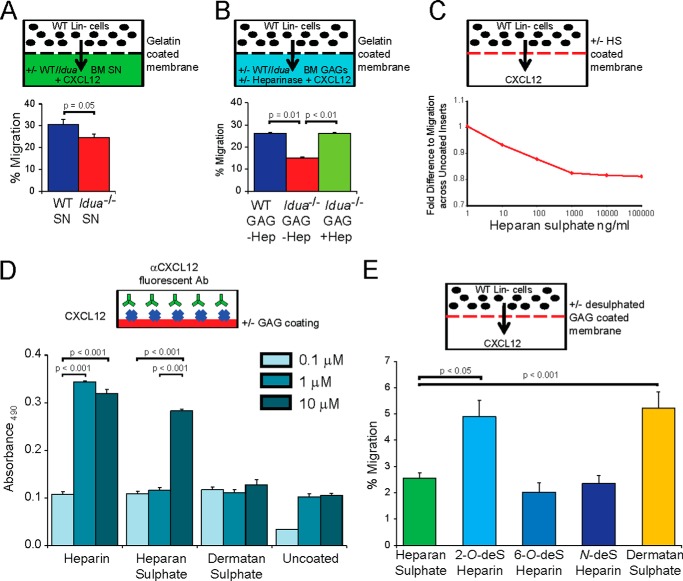
**Extracellular and 2-O sulfated HS in MPSI inhibits migration in a dose dependent manner.** *A*, transwell migration of WT Lin- cells toward exogenous CXCL12 added to the soluble fraction of WT or *Idua*^−/−^ BM (*n* = 8). Negative control of WT soluble fraction without exogenous CXCL12 is equivalent to 7.1% *B*, transwell migration of WT Lin- cells toward exogenous CXCL12 added to the Pronase treated glycosaminoglycan fraction of WT or *Idua*^−/−^ BM (*n* = 20) with or without heparinase pre-treatment. *C*, representative curve of WT BM migrated toward CXCL12 across HS-coated inserts at increasing concentrations (*n* = 4). *D*, comparison of CXCL12 binding to increasing concentrations of immobilized GAGs (*n* = 3). *E*, transwell migration of Lin- WT cells across membranes coated with 15 μm HS, DS and 2-*O*, 6-*O*, and *N*-desulfated heparin (*n* = 4). Positive control of uncoated membrane is equivalent to 11.2%. Error bars represent ± S.E.

The increase in HS in the bone marrow compartment suggested that excess HS may inhibit CXCL12 function in migration, in contrast to previous findings (12). Therefore we used a migration assay to determine if the HS produced by *Idua*^−/−^ animals was indeed inhibitory. In agreement with our *in vivo* data we established that the soluble extracellular fraction of *Idua*^−/−^ BM significantly reduced CXCL12-mediated transwell migration of lineage negative WT HSPCs, compared with WT supernatant (Fig. 6*A*). To eliminate the possibility of protein interactions, including CXCL12 saturation, being responsible for the inhibition of migration, GAGs chains were purified from *Idua*^−/−^ or WT BM, including a protease and boiling step to degrade/denature co-purified proteins. Purified *Idua*^−/−^ GAGs resulted in a similar reduction in CXCL12 mediated migration of WT HSPC to CXCL12 (Fig. 6*B*). Critically this effect was eliminated by pre-treating the purified GAGs with HS-degrading heparinases prior to transwell migration, ruling out involvement of excess DS or chondroitin sulfate, and confirming a role for HS in decreased HSPC migration (Fig. 6*B*).

##### Highly Sulfated HS and Heparin Bind CXCL12 in a Dose-dependent Manner

A dose-dependent decrease in CXCL12-mediated HSPC migration was also obtained when increasing amounts of HS were immobilized on the transwell migration membrane (Fig. 6*C*). Thus, suggesting that both soluble and immobilized HS can block migration. To demonstrate that HS was binding CXCL12 in a sulfation and dose dependent manner, GAG was immobilized on the membrane, incubated with CXCL12, washed, and then a CXCL12 antibody was used to probe for CXCL12-GAG binding. Only high concentrations of HS (10 μm) were effectively able to capture CXCL12. Heparin, which is a fully sulfated analog of HS, bound CXCL12 at lower concentrations than HS (Fig. 6*D*), whereas DS was unable to bind. This is in keeping with previous work (20) showing that CXCL12 preferentially binds to the sulfated regions of HS (which in HS are normally interspersed by regions of low sulfation modification).

##### 2-O-Desulfation of Heparin Rescues CXCL12-mediated Hematopoietic Migration

Finally, we coated transwell membranes with selectively desulfated heparin, to highlight the importance of different sulfate residues, and observed their effect on CXCL12-mediated migration of WT HSPC. HS, 6*-O*-desulfated and *N-*desulfated heparin reduced overall HSPC migration, mimicking the effect seen with *Idua*^−/−^ BM GAG fractions; both 2*-O-*desulfated heparin and DS had no inhibitory effect (Fig. 6*E*). Thus these data strongly suggest that 2-O-sulfation is essential for CXCL12 binding and excess 2-*O*-sulfated HS in *Idua*^−/−^ animals binds and functionally inhibits CXCL12-mediated HSPC migration, a modification which we found to be over-represented in *Idua*^−/−^ BM.

## DISCUSSION

The ability of most lysosomal enzymes to cross-correct neighboring cells via the mannose-6-phosphate pathway makes HSCT an attractive treatment option for lysosomal diseases. Despite this, HSCT is still only used for a handful of lysosomal diseases, including MPSI-H. Reduced intensity conditioning could significantly improve the safety of HSCT in MPSI-H, potentially broadening its scope to attenuated disease. In principle, stable mixed chimerism is all that should be required for cross-correction, however reduced intensity conditioning regimens are rarely successful, and the reasons for this are not well understood (5, 6). Previous studies have investigated the effect of HS/DS storage on developmental signaling pathways, however conclusions regarding the nature of the accumulated HS, and thus its effect on signaling pathways, have been contradictory (32, 34) or inconclusive (41). To date there have been no investigations into the possible effects of accumulated HS on pathways important for disease treatment. Here, we sought to determine whether the stored HS may have a functional role in HSPC migration and engraftment in MPSI-H, to enable a greater understanding of why reduced intensity conditioning HSCT is usually unsuccessful in the clinical setting.

Using the *Idua*^−/−^ mouse model of MPSI-H we demonstrated that there is a defect in both HSPC migration and engraftment in MPSI-H. However, interestingly the engraftment defect is only significant under limiting transplant conditions, while the migration defect is measurable even at saturating cell doses and full myeloablation. This suggests that the migration defect is constitutive but only contributes to graft failure under sub-optimal conditions for graft acceptance, where cell numbers are limiting, or where reduced intensity conditioning is used. The absence of an engraftment defect in non-limiting conditions, together with evidence that hematopoiesis and hemostasis in the *Idua*^−/−^ mouse are indistinguishable from WT, suggests a BM niche that is supportive of engraftment.

In challenge to the current understanding of lysosomal storage diseases, we demonstrated a 13-fold excess of HS in extracellular locations in the *Idua*^−/−^ BM niche, at least some of which is still bound to a protein core, presenting a system in which it is possible that multiple signaling pathways may be altered. Notably, the detection of non-reducing HS end structures in the plasma of patients with MPSI (42), suggests that HS is being partly degraded in the lysosome and released. The large excess of HS that we see in the ECM, existing most likely as a mixture of proteoglycan “bound” 3G10 positive cores and free 10E4 positive chains may be contributing to reduced mobility of HS in and out of cells. This results in accumulation in the ECM and consequential sequestration of cytokines and chemokines including CXCL12, thus altering critical signaling pathways.

**FIGURE 7.**
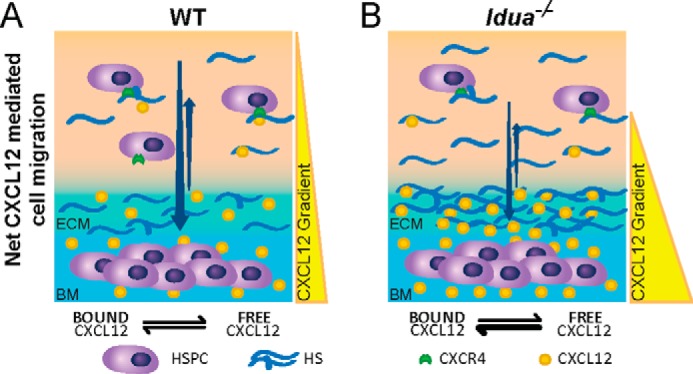
**Working model of HS mediated HSPC migration and engraftment defect in MPSI.** *A*, in normal WT mice CXCL12 is produced by the bone marrow and presented in a haptotactic gradient by HS. An equilibrium exists between HS bound and unbound CXCL12. HSPCs are mainly retained in the bone marrow by the CXCL12 gradient and some HS is required for CXCL12 dimerization and binding to CXCR4 receptors on HSPCs. Migration to bone marrow is reliant on increased CXLC12 production by bone marrow cells. *B*, bone marrow cells in *Idua*^−/−^ mice contain an excess of intra- and particularly extracellular HS that is highly sulfated and also elevated in the peripheral blood. Despite increased CXCL12 levels in the *Idua*^−/−^ bone marrow niche, increased binding of CXCL12 by HS and thus sequestration leads to increased retention of HSPCs within the bone marrow and reduced migration after myeloablation. Full intensity myeloablation may not increase cell numbers migrating over reduced intensity conditioning, but further damages the recipient niche giving donor cells a selective advantage within the niche despite the poor migratory environment present in MPSI.

CXCL12-mediated homing of HSPCs to the BM following transplantation relies on the formation of appropriate haptotactic gradients involving HS (Fig. 7). The requirement for HS in CXCL12-mediated migration is demonstrated in a study where mice expressed a mutant form of CXCL12 unable to bind HS. Increased circulating CXCL12 and reduced migration of CD34+ cells to sites of ischemic injury was observed due to an absence of haptotactic CXCL12 gradients (43). Furthermore, it has been shown that entry and egress of HSPCs to the bone marrow may be controlled through manipulations of HS. For instance, a number of studies (30, 31), have indicated that increases in circulating GAG mimetics lead to egress of CXCL12 from the BM niche into circulation, and a concomitant mobilization of circulating HSPCs. We have previously shown elevated HS in the circulation of the *Idua*^−/−^ mouse and MPSI patients (44, 45), which might be expected to behave in the same way as circulating GAG mimetics, increasing HSPC egress from the BM. However, within the *Idua*^−/−^ mouse, the significant increase in BM niche HS is conversely mitigating the effect of circulating HS, essentially trapping CXCL12 in the BM niche, and consequently decreasing circulating HSPCs. The CXCL12 gradients presented in Fig. 7 might suggest that migration would be normal in the *Idua*^−/−^ mouse (CXCL12 being at the highest concentration where HS is most localized, in the BM), and possibly even better than WT, given the higher concentration of CXCL12. However, our observations, together with previous studies on the affinity of CXCL12 for *2-O*-sulfated HS, suggest that high-affinity binding of CXCL12 by heavily sulfated *Idua*^−/−^ HS is preventing CXCL12 from forming effective haptotactic gradients, resulting in sequestration of CXCL12 in the BM (Fig. 7*B*).

Although full intensity busulfan conditioning, does not increase the numbers of donor HSPC reaching the BM (46), it does provide a more permissive environment for engraftment of donor HSPC by eliminating a greater proportion of recipient BM, thus creating niche space. The defect in HSPC migration and excess of GAG in *Idua*^−/−^ BM strongly suggest that HSCT in HS storage diseases such as MPSI-H should use full intensity conditioning, and provide a mechanism by which this strategy is successful in improving MPSI patient engraftment outcomes (5, 7). Our data also suggest that strategies such as ERT, which reduce the amount of HS in and around cells prior to transplant, may improve donor chimerism outcomes by reducing HS-mediated homing inhibition in patients. MPSI-H patients are known to raise significant inhibitory antibody responses to ERT that are subsequently abolished by successful HSCT (39). Thus, abbreviated pre-transplant ERT regimens may be preferable to avoid inactivation of delivered enzyme prior to HSCT to maximize outcomes, and this warrants further investigation.

In conclusion, we show that highly 2*-O*-sulfated HS is present in excess in extracellular locations in the BM in MPSI-H, and in this context has an unexpected role in inhibiting HSPC migration into, and out of, the BM by sequestering CXCL12. Our data further define the functional role of HS in CXLC12-mediated HSPC migration and suggest a viable treatment approach for achieving successful outcomes with HSCT after reduced intensity conditioning in MPSI-H.

## Footnotes

2 The abbreviations used are:

MPSI: mucopolysaccharidosis type I
MPSI-H: Hurler disease
GAG: glycosaminoglycans
HS: heparan sulfate
DS: dermatan sulfate
HSCT: hematopoietic stem cell transplantation
HSPC: hematopoietic stem and progenitor cell
BM: bone marrow
Idua: α-l-iduronidase
ERT: enzyme replacement therapy
HSPG: HS proteoglycan
GlcNAc: *N*-acetyl glucosamine
GlcA: glucuronic acid
IdoA: iduronic acid
AMAC: 2-aminoacridone
CFU: colony-forming units.
